# A review of methods for assessment of cognitive function in high‐altitude hypoxic environments

**DOI:** 10.1002/brb3.3418

**Published:** 2024-02-26

**Authors:** Haojie Fan, Ying Meng, Lingling Zhu, Ming Fan, Du‐Ming Wang, Yong‐Qi Zhao

**Affiliations:** ^1^ Department of Psychology Zhejiang Sci‐Tech University Hangzhou China; ^2^ Department of Cognitive and Stress Beijing Institute of Basic Medical Sciences Beijing China; ^3^ School of Information Sciences & Engineering Lanzhou University Lanzhou China

**Keywords:** altitude hypoxia, cognitive, cognitive assessment method, cognitive assessment system

## Abstract

Hypoxic environments like those present at high altitudes may negatively affect brain function. Varying levels of hypoxia, whether acute or chronic, are previously shown to impair cognitive function in humans. Assessment and prevention of such cognitive impairment require detection of cognitive changes and impairment using specific cognitive function assessment tools. This paper summarizes the findings of previous research, outlines the methods for cognitive function assessment used at a high altitude, elaborates the need to develop standardized and systematic cognitive function assessment tools for high‐altitude hypoxia environments.

## 1 BACKGROUND

With the development of economy and tourism, an increasing number of people from low altitudes choose to travel, work, or live in high‐altitude areas (Huang, 2021). Personnel on special assignments, such as emergency medical services, law enforcement and the military, often need to ascend rapidly to high altitudes and then quickly perform physically demanding tasks (Rodway & Muza, 2011). From military deployments to high‐altitude mining operations, the consequent lack of oxygen can pose a serious risk to health and an occupational hazard (Walsh et al., 2020). In high‐altitude areas, the lower air pressure causes oxygen particles to be more spread out. Therefore, when people breathe in, they encounter inadequate oxygenation in their blood and lungs (Luo et al., 2023). Even though the brain constitutes only around 2% of the body's weight, it utilizes 20% of the oxygen the body takes in (Raichle & Gusnard, 2002). Consequently, this situation results in an inadequate oxygen supply to the brains of individuals residing in high‐altitude regions.

Exposure to hypobaric hypoxia may elicit negative effects on the brain including damage to the structural integrity, perturbations to metabolism etc. (Forsberg & Herlenius, 2019). The severity of the effects of high altitudes on cognitive function is related to altitude and duration of hypoxic exposure (Yan, 2014). The mainstream view is that cognitive abilities are basically “highly dependent” on the altitude environment, that is, the higher the altitude, the more severe the impairment of cognitive functions, and the trend of changes in different cognitive functions is different (Wilson et al., 2009). Long‐term exposure to high altitude may lead to impairments on conflict control (Tulek et al., 2013). Moreover, attentional resources to resist the conflict control were reduced in the high‐altitude group (Ma et al., 2015). Studies have reported that healthy adult subjects with acute exposure to 6096 m for 15 min impairment in learning and memory, including the ability to encode, retrieve, and retain memory (Nation et al., 2017). Narrative evaluations have shown that acute and chronic hypoxia exposure may negatively affect various cognitive parameters such as attention and executive function (Virue ´s‐Ortega et al., 2004). While these studies assess the impact of high‐altitude hypoxic environments on cognitive function, limitations such as variations in the altitude and duration of exposure in the conducted experiments, as well as differences in the measurement tools chosen for assessing the same cognitive functions, may affect the generalizability of the results. At the same time, the categorization of test procedures into cognitive domains lacks clear, and objective criteria and might vary across studies, so, in order to assess cognitive function during missions and emergency procedures in high‐altitude environments, it is crucial to establish a standardized framework for tests to ensure consistency across various studies. Research related to high‐altitude hypoxia and cognitive function is generally on the rise, with hot research areas focusing on “sleep,” “acclimatization,” and “cognitive impairment.” The research direction is concerned with the effects of environments and exposure time on individuals and how to prevent or recover such negative effects, while the research on cognitive function assessment methods in high‐altitude environments is still insufficient. Therefore, this paper summarizes previous studies from the perspective of cognitive assessment and compares cognitive assessment methods and tools used in the high‐altitude (hypoxia). We explore the improvement direction of cognitive function assessment tools for high altitudes, provide reference for the development of cognitive function assessment system in high altitudes, and make a prospect for the development of new tools and techniques in the future.

## 2 LITERATURE SEARCH AND SELECTION (METHOD)

### 2.1 Search strategy

The study conducted a systematic literature review to explore the methods for assessment of cognitive function in high‐altitude hypoxic environments, adhering to the guidelines outlined in the Preferred Reporting Items for Systematic Reviews and Meta‐Analysis (PRISMA).

We took a systematic approach by leveraging previous systematic reviews (Martin et al., 2019), in order to develop a robust search strategy for our study. A search of Medline (PubMed) and Web of Science was then conducted up to April 2022, using (“normobaric hypoxia” OR “hypobaric hypoxia” OR “altitude”) AND (“Mental Processes” [Mesh]) in “all fields” for PubMed; and using (altitude OR “normobaric hypoxia” OR “hypobaric hypoxia”) combined with (“cognition” OR “cognitive”) in “all fields” for Web of Science and EMBASE.

Initially, all titles of the returned articles were examined. The purpose of this screening was to identify and exclude any duplicate articles or those that were clearly unrelated to the research topic. Duplicates or studies that were deemed nonrelevant were eliminated from further consideration. After identifying articles through electronic databases, we manually checked those articles for any citations referencing other relevant articles that may not have been captured in the initial electronic search. At the same time, only focusing on English language, peer‐reviewed studies.

### 2.2 Inclusion and exclusion criteria

Studies were included from the initial review of articles if they met the following criteria: (a) included an adult population without injury, neurological condition, or mental illness which may affect the cognitive performance of the participant; (b) the primary aim of the study was to assess the impact of hypoxia on cognitive or task performance; and (c) included a control condition, or graded levels of the intervention (e.g., the sea level vs. 3000 vs. 4000 m altitude).

**FIGURE 1:**
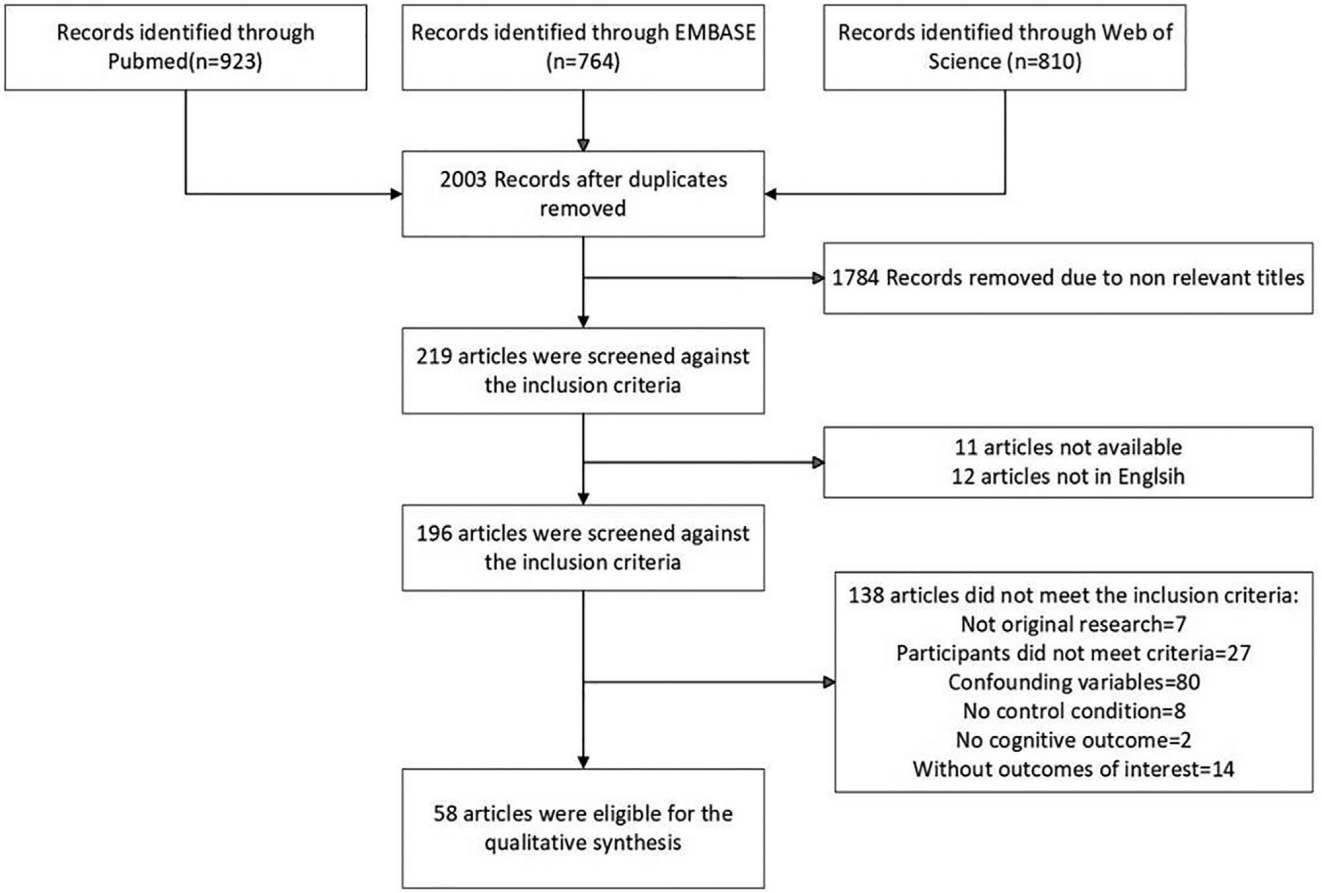
Flow diagram of study selection.

Studies were excluded: (a) if the impact of a hypoxia environment was confounded or mixed with the effects of another variable. Specifically, variables such as exercise, sleep deprivation, or using drugs were mentioned as examples; (b) studies that have been carried out with individuals who are professional pilots or military personnel; and (c) studies that exclusively utilize electrophysiological methods to assess cognitive processes (e.g., ERP). Flow diagram of study selection was showed as Figure 1.

## 3 RESULTS

### 3.1 Study selection

The initial search returned a total of 2497 records; after removing duplicates, there were 2003 remaining articles. Subsequently, we screened the titles and abstracts of these articles and found that 1784 records were irrelevant to our research topic. Following the removal of articles that were inaccessible or not in English, we applied the inclusion criteria to the remaining 196 articles and identified that 138 studies did not meet the inclusion criteria, specifically: not original research (7), participants did not meet criteria (27), confounding variables (80), no control group (8), no cognitive outcome (2), without outcome of interest (14). Finally, 58 articles were included in our analysis.

A total of 117 different neuropsychological tests in 8 different cognitive domains: sensation, perception, motor skills and construction, attention and concentration, memory, execution function, processing speed, language/verbal skills. The current cognitive function assessment methods can be roughly divided into three categories according to the operation methods: traditional questionnaires, classical experimental paradigm, and computerized cognitive assessment systems.

### 3.2 Traditional questionnaires and scales

The refinement of classical psychometric theories has created a standardized process for psychometric test development, giving rise to a large number of neuropsychological tests that are the primary means of early cognitive assessment.

#### 3.2.1 Intelligence test

The Wechsler Adult Intelligence Scale (WAIS), developed and published by the American psychologist Wechsler in 1955, measures the intelligence of adults aged 16 to 74. Its revised version (WAIS‐R) was published in 1981, including two parts of verbal and operational scales. Recommended by Chinese psychologists in the early 1980s, Gong presided over the publication of a localized WAIS China Revision (WAIS‐RC) based on the Chinese cultural background, which established two sets of normal, rural, and urban, including six verbal subtests and five operational subtests (Dai et al., 1999).

In studies, researchers may select different subtests to assess specific cognitive functions in a targeted manner depending on the purpose of the experiment. A study (Zhang et al., 2011) using Wechsler Memory Scale (Gong et al., 1989), a subset scale of WAIS, measures new verbal learning and visual and verbal memory functions of participants who are living for long term at moderate altitude. The study found that the subjects at moderate altitudes performed significantly worse than the sea level controls in short‐term visual construction. This means that when it came to tasks related to visualizing and reproducing objects or designs in the short term, the individuals at moderate altitudes demonstrated lower performance compared to the sea level.

#### 3.2.2 Raven Standard Progressive Matrices

Raven's Standard Progressive Matrices (RSPM) is highly regarded as one of the most popular and extensively utilized psychometric tests for assessing general cognitive abilities according to the procedure suggested by (Mackintosh, 1998). It consisted of five sets labeled A to E, each containing 12 items (for example, A1 through A12). The items within each set progressively increased in difficulty, requiring higher levels of cognitive abilities to encode and analyze information. The scoring of the test was based on two factors: the duration of completion and the total number of correct attempts.

Sharma et al. (2014) conducted on 761 male volunteers who were between the ages of 25 and 35 years. These participants had never been to high altitudes before. Then the volunteers were taken to an altitude range of 4200–4600 m above mean sea level and stayed there for 18 months. Compared to the baseline, the exposure of high altitude revealed an impairment in executive functioning among the participants.

#### 3.2.3 Cognitive impairment screening scale

The Mini‐Mental State Examination (MMSE) is well‐established assessment scales used for screening or grading dementia and various cognitive disorders, with systematic evaluations showing that the MMSE meet all psychometric and clinical utility criteria for any level of cognitive impairment and have been widely used among clinical patients (Burton & Tyson, 2015). The MMSE with 60% of its components related to memory and language functions and has a higher accuracy rate for screening for poststroke dementia but is less effective for screening for mild cognitive impairment (Guo et al., 2000).

Sharma et al. (2014) use The Mini‐Mental State Examination (MMSE) to test the effect of prolong stayed at high altitude, as the duration of the stay at high altitude increased, the proportion of participants exhibiting MCI also increased. Specifically, the prevalence of MCI rose from 8.17% after 3 months of stay to 18.54% after 18 months of stay. Furthermore, result indicates that there were impairments in various cognitive domains associated with visuospatial executive function, attention, delayed recall, and procedural memory. These cognitive domains were affected following the prolonged stay at high altitude.

### 3.3 Classical experimental paradigm

The experimental paradigm refers to the classical experimental tasks or relatively fixed experimental procedures in the study of various psychological processes and various branches of psychology. In principle, there are many ways to explain any psychological phenomenon. One of the direct reasons for this confusion is that the input and output factors, external and internal factors of cognitive experiments are not all visible and controllable. The experimental paradigm can be designed to manipulate various variables and conditions for different cognitive problems, to control the exclusion of invisible factors, to separate confounding factors, and to reveal phenomena and mechanisms beyond common knowledge. Cognitive science and experimental psychologist Chen et al. (2017) stated that the cognitive experimental paradigm is one of the three foundations of cognitive science and a scientific experimental protocol suitable for the study of cognitive processes. A researcher may select an appropriate experimental paradigm or combine multiple paradigms for assessment, depending on the content of the study.

This paper focuses on human exposure to hypoxia and categorizes the common experimental paradigms used when measuring different cognitive functions under this condition, as shown in Table 1. It identified 107 distinct neuropsychological tests, and these tests were categorized into seven different cognitive domains. And among these tests, the Stroop Test is the most commonly utilized. We also list studies that have investigated the effects of altitude on cognitive performance. An overview of these studies is presented in Table S1.

**TABLE 1:** Cognitive tasks and cognitive task categories.

Category	Task	Measurement content	Reference
Sensation	King–Devick Test	Visual	Stepanek et al. (2013)
Perception	Line Bisection Test	Visual and spatial perception	Merz et al. (2013)
	Line Orientation Task		Petiet et al. (1988), Frost et al. (2021)
	Abstract Matching Task		Frost et al. (2021)
	Spatial Discrimination		Subudhi et al. (2014)
	Reading of Briefly Displayed Letters		Schlaepfer et al. (1992)
	Time Wall Estimation Task	Time perception	De Bels et al. (2019)
	Time Perception Task		Davranche et al. (2016)
Motor skills and construction	Pegboard–Psychomotor Test	Psychomotor ability	Abraini et al. (1998), Merz et al. (2013), Griva et al. (2017)
	Finger–Tapping Task		Petiet et al. (1988), Kramer et al. (1993), Turner et al. (2015)
	Santa Ana Manual Dexterity Test		Zhang et al. (2013)
	Pursuit Aiming Test		Zhang et al. (2013)
	Visual Motor Reaction Time		Weigle et al. (2007)
	Psychomotor Speed Task		Pighin et al. (2012)
	Benton Visual Retention Test	Coping	Zhang et al. (2013)
	The Rey–Osterrieth Complex Figure	Coping	Zhang et al. (2011)
	Clock Drawing Test (CDT)	Drawing	Sharma et al. (2014)
	Bender Visual Motor Gestalt Test		Sharma et al. (2014)
	Motor Praxis Task		Frost et al. (2021)
	Block Design	Praxis skills (assembling and building, etc.)	Griva et al. (2017)
Attention and concentration	Trail making A Task		Asmaro et al. (2013), Sharma et al. (2014), Griva et al. (2017), Issa et al. (2016), Latshang et al. (2013), Pun, Hartmann et al. (2018)
	Modified Continuous Performance Task	Complex attentional functions	Altbäcker et al. (2019)
	Digit Symbol Substitution Test		Harris et al. (2009), Frost et al. (2021), Falla et al. (2021), Zhang et al. (2013), Turner et al. (2015)
	Divided Attention Steering Simulator		Latshang et al. (2013)
	Code Substitution Task		Kramer et al. (1993)
	Paced Auditory Serial Addition Test		Malle et al. (2016), Petiet et al. (1988), Shi et al. (2016)
	Ruff 2/7 Cancellation Test		Merz et al. (2013)
	Selective Auditory Attention Task		Petiet et al. (1988)
	Paced Visual Serial Addition Test		Shi et al. (2016)
	Eriksen flanker		Williams et al. (2019)
	Letter Cancellation Test		Griva et al. (2017)
	Symbol Digit Modalities test		Griva et al. (2017)
Attention and concentration	Colorado Perceptual Speed Test		Karinen and Tuomisto (2017)
	Visual search task		Zhang et al. (2018)
	Flanker Task		Lefferts et al. (2019) Ma et al. (2019a,b,c)
	Psychomotor Vigilance Test	Vigilance	Thomas et al. (2007), Latshang et al. (2013), Falla et al. (2021), Frost et al. (2021), Pun, Hartmann et al. (2018)
	Perceptual Vigilance Task	Sustained attention	De Bels et al. (2019)
	Monitoring Task Reaction Time		Harris et al. (2009)
	Monitoring Task Accuracy		Harris et al. (2009)
	Divided Attention Steering Simulator		Latshang et al. (2013)
	Frankfurt Attention Inventory‐2		Limmer and Platen (2018)
	Rapid Visual Processing Test		Pun, Guadagni et al. (2018)
	Attention Switching Task		Pun, Guadagni et al. (2018), Turner et al. (2015)
	Working Memory Task Reaction Time		Harris et al. (2009)
	Continuous Performance Test		Turner et al. (2015)
Memory	Corsi Block Forwards	Short‐term memory	De Aquino Lemos et al. (2012)
	Digit Span Test Forward		Harris et al. (2009), Petiet et al. (1988), Malle et al. (2016), De Aquino Lemos et al. (2012), Asmaro et al. (2013), Shi et al. (2016), Zhang et al. (2013), Subudhi et al. (2014)
	Picture Recall Test		Shi et al. (2016)
	Picture Recognition Test		Shi et al. (2016)
	Pocket Calculator Cognitive Motor Task		Bonnon et al. (1999)
	Code Substitution delayed recall		Subudhi et al. (2014)
	Memory Search Task		Kramer et al. (1993)
	Verbal Free Recall		Pelamatti et al. (2003)
	Word Span Forward		Phillips and Pace (1966)
	Number Recall Test		Crow and Kelman (1971)
	Corsi Blocks Backwards	Working memory	De Aquino Lemos et al. (2012)
	Digit Span Test Backwards		Harris et al. (2009), Malle et al. (2016), Asmaro et al. (2013) De Aquino Lemos et al. (2012), Zhang et al. (2013)
	Verbal Working Memory Capacity		Parker et al. (2017)
	Random Number Generation		De Aquino Lemos et al. (2012)
	Sequence of Numbers and Letters		De Aquino Lemos et al. (2012)
	Working Memory Task Accuracy		Harris et al. (2009)
	Memory Interference Task		Loprinzi et al. (2019)
	Running Memory Continuous Performance Test		Seo et al. (2015), Seo et al. (2017)
	Mental Rotation		
	Sternberg's Memory Search		Subudhi et al. (2014)
	N‐back		Ma et al. (2019a,b,c), Ma (2020), Zhang et al. (2011) Latshang et al. (2013), Thomas et al. (2007), Lefferts et al. (2019) Williams et al. (2019), Frost et al. (2021)
	Rey's Auditory–Verbal Learning Test	Verbal memory	Harris et al. (2009), Griva et al. (2017), Zhang et al. (2011)
	Selective Reminding Test		Petiet et al. (1988)
	Verbal Memory Test		Chen et al. (2017), Turner et al. (2015), Xin et al. (2020)
	Serial Digit Learning Test		Sharma et al. (2014)
	Visual Object Learning Task	Visual memory	Frost et al. (2021)
	Match to sample		Subudhi et al. (2014)
	Learning Task Accuracy		Harris et al. (2009)
	visual memory test		Chen et al. (2017), Turner et al. (2015), Xin et al. (2020)
	category search task	Semantic memory	Kramer et al. (1993)
	Word Free Recall	Long‐term explicit memory	Zhang et al. (2011)
	Degraded picture recognition	Implicit memory	Zhang et al. (2011)
Execution function	Gorham's Proverbs	Reasoning	Petiet et al. (1988)
	Modified Math Processing Task		De Bels et al. (2019)
	Robinson's Numbers tests		Phillips and Pace (1966)
	Number Ordination—Rey's test		Abraini et al. (1998)
	Balloon Analogue Risk Taking task	Decision‐making	Pighin et al. (2014), Pighin et al. (2020), Falla et al. (2021), Frost et al. (2021)
	The Financial Decision‐Making Task		Pighin et al. (2012)
	Controlled Oral Word Association		Harris et al. (2009), Griva et al. (2017)
	Choice Reaction Time		Pramsohler et al. (2017), Kramer et al. (1993), Harris et al. (2009)
	Four‐Choice Reaction Time		Dykiert et al. (2010)
	Number Comparison Test		Karinen and Tuomisto (2017)
	Rapid Cognitive Assessment Tool		Issa et al. (2016)
	Ruff Figural Fluency Test		Merz et al. (2013)
	Simon Task		Davranche et al. (2016)
	Stroop Test		Griva et al. (2017), Lefferts et al. (2020), Issa et al. (2016), De Aquino Lemos et al. (2012), Ochi et al. (2018), Asmaro et al. (2013), Altbäcker et al. (2019), Sharma et al. (2014), Turner et al. (2015), Weigle et al. (2007)
	Trail Making Test B		Griva et al. (2017), Pun, Hartmann et al. (2018), Harris et al. (2009), Issa et al. (2016), Asmaro et al. (2013), Sharma et al. (2014)
	Go/No‐Go Test	Inhibitory control	Chen et al. (2017), Seo et al. (2015), Subudhi et al. (2014), Xin et al. (2020)
	Two‐choice Oddball Task		Wang et al. (2021)
	Pro‐point and Anti‐Point Tasks		Gibbons et al. (2020)
	One Touch Stockings of Cambridge Task		Pun, Guadagni et al. (2018)
	Visual Choice Reaction Time		Abraini et al. (1998)
Processing speed	Deary–Liewald Reaction Time Task		Williams et al. (2019)
	Pattern Comparison Task		Kramer et al. (1993)
	Procedural Reaction Time		Subudhi et al. (2014)
	Serial Reaction Time Task		Zhang et al. (2011)
	Simple Reaction Time Test		Zhang et al. (2013), Chen et al. (2017), Harris et al. (2009), Pun, Guadagni et al. (2018), Roach et al. (2014), Subudhi et al. (2014), Xin et al. (2020)
Language/verbal skills	Robinson's Rhymes tests		
	Category Fluency Tasks		Pavlicek et al. (2005)
	Verbal Letter Fluency		Pavlicek et al. (2005), Sharma et al. (2014)
	Verbal Reasoning Test		Weigle et al. (2007)
	Boston Naming Test		Petiet et al. (1988)

#### 3.3.1 Sensation and perception

Sensation refers to the process of detecting and registering stimuli from the environment through our senses (Harvey, 2019). One study conducted by Stepanek et al. (2013) examined the visual sensation through King–Devick Test.

In the domain of perception, sensory information is received, processed, and integrated. One of the key aspects of perception involves the ability to identify or recognize objects, sounds, or other stimuli based on previously experienced information. Perception can be evaluated by assessing an individual's capacity to recognize and correctly identify objects or sounds, as well as by examining the integrity or completeness of their perceptual fields (Harvey, 2019). Seven different tests were used to measure visual and spatial perception and time perception. Visual and spatial perceptions are the most frequently examined perceptual domains in altitude or hypoxia environment.

#### 3.3.2 Motor skills and construction

Fine motor skills like manual dexterity and broader abilities like balance make up the fundamental building blocks of human motor activity. Assessing each of these basic elements provides insight into an individual's overall motor functioning, capabilities, and control; construction refers to a domain of visual–spatial and motor skills involved in the reproduction and formation of basic drawings, either via copying or from memory (Harvey, 2019). Psychomotor abilities are the skills most commonly assessed within the domains of motor skills and constructional abilities. Regarding the field tests, Pegboard‐Psychomotor Test and Finger‐Tapping Task are the most conducted to examine psychomotor ability.

#### 3.3.3 Attention and concentration

Attention and concentration are broad mental skills that include both the capacity to selectively focus on target information and ignore distractions, as well as the vigilance to maintain that focused attention consistently over time (Harvey, 2019). Twenty‐five different tests were performed to examine the effects of hypoxia on attention and concentration. Among these tests, Trail making A Task and Psychomotor Vigilance Test methods are the most commonly used.

#### 3.3.4 Memory

Efficient memory is not only about initially encoding and storing information effectively but also about the ability to maintain that information even when challenges or distractions are introduced. Memory is a complex construct made up of many subcomponents, and rigorous assessment of memory requires using a wide array of validated and specially designed tests for each part. Two subcategories of memory, short‐term memory and working memory, have garnered the attention of researchers. For short‐term memory, 10 different tests were examined the effects of high‐altitude environment. Digit Span Test Forward test is the most frequently utilized; for working memory, 11 different tests were performed. “N‐back test” is the assessment tool most commonly employed to evaluate or assess an individual's working memory.

#### 3.3.5 Execution function

This cognitive domain is often known as reasoning and problem solving. It is a comprehensive concept that involves processes responsible for controlling and optimizing other cognitive abilities. This control enables individuals to efficiently address problems and make plans for the future. Executive functioning is critical for higher‐level cognitive functions, decision‐making, and goal‐oriented tasks (Harvey, 2019). Twenty different tests were used to examine this cognitive function. Stroop tests were performed in 11 studies, which is commonly utilized to assess executive function.

#### 3.3.6 Processing speed

Processing speed measures evaluate the rapidity of cognitive operations across tasks ranging from simple to complex. In processing speed tasks, participants are directed to solve the given activities as quickly as possible (Harvey, 2019). Five distinct tests were conducted to assess processing speed function. Simple reaction time tasks were the predominant measure used to evaluate processing speed capacities.

#### 3.3.7 Language/verbal skills

Language skills encompass receptive and expressive capacities, including comprehension, semantic access, objects naming, and following verbal instructions through actions (Harvey, 2019). Language skills are evaluated through measures of verbal fluency (e.g., naming as many animals as possible), object naming tasks, and tests requiring responses to instructions. Five discrete tests assessed language skills.

### 3.4 Computer test batteries

Currently, neurocognitive assessments are gradually changing from the previous paper‐and‐pencil scale tests and operational tests to computerized assessments (Zhang et al., 2020). Computerized assessment systems that program tests in cognitive psychology into a computer, it allows for strict control of stimulus presentation, precise measurement of response times, reduction of manipulation and recording errors due to subjective factors of the experimenters, and improved standardization of the test. Using computers, digital tablets, handheld devices or other digital interface management systems, a large number of individuals can be quickly tested, with the advantages of ready evaluation, accurate measurement, short‐time consumption, low cost, easy management in different languages, automatic export of data, no professional involvement, automatic calculation of results and generation of descriptive summaries of test results (Bauer et al., 2012).

#### 3.4.1 CNSVS

CNS Vital Signs (CNSVS) is a computerized neurocognitive test battery developed specifically for routine clinical screening purposes, developed by Gualtieri and Johnson (2006). It is a standardized, automated, and concise computerized battery neuropsychological tests which consists of seven tests: verbal and visual memory, finger tapping, symbol digit coding, the Stroop Test, a test of shifting attention and a continuous performance test. Chen et al. (2017) aim to examine and understand the changes that occur in both cognitive processes (such as attention, memory, and executive functions) and brain structure/function as a result of chronic high‐altitude exposure. Using the CNSVS, it is found that participants who were immigrating to Tibet for 2 years showed both decreased accuracy in memory tests and longer reaction times. The study found evidence of impaired working memory and psychomotor function during chronic high‐altitude exposure. About the acute exposure. used CNSVS to evaluate neurocognitive performance after the participants experienced the exposure at 5500 m by breathing a gas mixture. Exposure to hypoxia had a substantial negative impact on neurocognitive performance across multiple domains. The significant declines in performance indicate that hypoxia had detrimental effects on cognitive abilities such as memory, processing speed, attention, and executive function

#### 3.4.2 CogState

CogState is a computerized cognitive test battery designed for assessing changes in cognitive function. This test has been shown to possess favorable psychometric properties, making it a reliable tool for repeated or serial studies focusing on cognitive assessment (Collie et al., 2001; Makdissi et al., 2001). It is designed to be easily administered by nonexperts, making it accessible for use by a broader range of individuals and the test's sensitivity in detecting and assessing mild cognitive impairment, regardless of the underlying factors or causes contributing to the impairment (Darby et al., 2002; Silbert et al., 2004; Snyder et al., 2005). CogState brief battery covers a range of cognitive functions such as reaction time, attention, working memory, decision‐making, and visual learning. These tasks collectively aim to provide a comprehensive assessment of cognitive abilities within a condensed testing format. Harris et al. (2009) used the test battery focusing on assessing cognitive impairment in a typical commercial trekking cohort after a gradual ascent to 5100 m over an 18‐day period. Although the group experienced a decline in monitoring test reaction time at an altitude of 5100 m, other cognitive functions do not show significant changes.

#### 3.4.3 Joggle Research Battery

The Joggle Research Battery uses an interactive, brief, game‐like format on the iPad, making it engaging and potentially more enjoyable for the participants. It tracks performance across these eight tasks: Motor Praxis, Visual Object Learning, N‐Back, Abstract Matching, Line Orientation, Digit Symbol Substitution, Balloon Analog Risk, and Psychomotor Vigilance, providing valuable insights into various cognitive domains including sensory motor speed, attention, visual learning and memory, working memory, decision‐making, abstraction, and spatial orientation (Gamaldo et al., 2020). In a field study, Frost et al. (2021) examined the effect of exposure at 3800 m over 3 days. Results show that there were differential effects of high‐altitude exposure on reaction times. The psychomotor vigilance task showed slower reaction times at high altitude without improvement, while the balloon analog risk task showed decreased reaction times at high altitude and maybe indicating increased risk‐taking behavior.

#### 3.4.4 DANA

The Defense Automated Neurobehavioral Assessment (DANA) tool was used to assess neurocognitive function. DANA includes a library of standardized cognitive and psychological assessments and is administered using a handheld computer or similar portable device (Lathan et al., 2013). Its purpose is to provide a reliable and standardized method for evaluating neurocognitive performance. This test battery, which consists of 9 tests, covers a wide range of cognitive domains. In a field study, Subudhi et al. (2014) evaluate the effect of exposure at 5260 m during 16 days. Researchers find that compared to sea level, the assessment at day 1 shows longer reaction time in simple reaction task, Choice reaction time task, but a decrease in Code substitution and Match to sample. However, all the tasks above mentioned improved by day 16. There were no significant changes in other tasks.

#### 3.4.5 ANAM

The Automated Neuropsychological Assessment Metrics (ANAM) is an automated neuropsychological assessment tool developed by the US Department of Defense. It is a sensitive and reproducible measure that has been used as a measure of cognition in clinical and related settings such as training exercises, chemical or radiation exposure, fatigue, brain injury, hypoxia heat stress, anxiety, metabolic difficulties, and related areas such as Parkinson's disease and Alzheimer's disease (Friedl et al., 2007; Lowe et al., 2007; Wilken et al., 2007). The test takes approximately 30 min and consists of seven tests: choice response time, tower test, continuous performance test, syntactic reasoning, arithmetic, sample match, spatial processes, and orientation. Some researchers have also used the ANAM to compare cognitive function during short‐term exposures of 30 min to 2 h at four altitudes: ground, 1524 m, 2438 m, and 3658 m. Finally, only continuous performance and grammatical reasoning task performance were found to be slightly reduced at the two highest altitudes, while 18 of the 33 possible symptoms were more common at 3658 m than at ground levels in the subjective symptom survey. The findings indicated that mild hypoxia had little influence on cognition compared to some of the classic symptoms of hypoxia (Pilmanis et al., 2016). Another study (Figueiredo et al., 2022) where 20 lowlanders who were not acclimatized to high altitude were exposed to either 3000 m or 4050 m for a duration of 20 h. The exposure was conducted in a hypobaric chamber. Results demonstrate that there was no significant difference between sea level group and high‐altitude group in cognitive performance.

#### 3.4.6 CANTAB

The Cambridge Automated Neuropsychological Assessment System (CANTAB) includes four assessment systems for core cognition, depression, ADHD, and dementia, with a total of 22 assessment tasks (Hodges & Xiong, 2013). CANTAB is suitable for both normal and psychiatric patients and has been widely used in academic research, drug clinical experiments, and healthcare. The core cognitive system measures situational memory, executive function, working memory, and reaction time, and researchers can select the items to administer as needed. For example, Pun et al. (2018) assembled a cognitive assessment system using a reaction time (RTI) task, an attentional switching (AST) task, and a rapid visual processing (RVP) task, combined with the One Touch stockings (OTS) task in the CANTAB system. The participants were exposed to the 5050 m altitude for 1 week each in two sessions and returned to low altitudes for 1‐week rest between exposures, and their processing speed, sustained attention, and executive function levels were measured by the cognitive measurement system during the two exposure cycles for the purpose of investigating the effects of acute, subacute (postadaptation), and repeated exposure to 5050 m altitude on cognition. It was found that sustained and selective attention was impaired at high altitudes and improved with adaptation, but that adaptive cognitive improvements obtained during the first exposure did not carry over to subsequent repeated exposures (Pun et al., 2018).

## 4 DISCUSSION

This paper's purpose is to conduct a review of the methods used to assess cognitive function in the current high‐altitude hypoxic environment. Based on our review, the primary discoveries from the present analysis indicate that a total of 107 distinct tests were employed. Attention and concentration, memory, and executive functions were the cognitive domains that received the most extensive research focus. Nevertheless, it is not possible to definitively assert that these domains were more sensitive than others, as impairments were observed across all cognitive domains. Of all the cognitive assessment tools utilized, the most commonly administered were the Stroop Test, the Digit Span Test Forward, and the N‐Back task. Same as cognition domains mentioned above, while the Stroop Test, Digit Span Forward, and N‐Back task were the most commonly used, conclusive evidence that they were more sensitive for detecting impairments compared to other methods is lacking, given that cognitive deficits were found with all assessment tools utilized.

### 4.1 Major findings

We discovered that the assessment methods can be categorized into three broad types or categories: traditional questionnaires and scales, classical experimental paradigm, computerized cognitive assessment systems.

Traditional questionnaires and scales primarily rely on intelligence scales and cognitive impairment screening scales to measure the effects of high‐altitude hypoxia on cognitive function. However, because of strict requirements for assessors, the time‐consuming and labor‐intensive nature of the testing process, the monotonous and unvaried format, and the difficulty in storing and managing textual data, these limitations underscore the need for alternative assessment methods that address these issues and provide more efficient and effective evaluations of cognitive function in such environments.

Among various methods used for assessing cognitive function in high‐altitude environments, the most commonly employed and widely accepted approach is the utilization of classic experimental paradigms. These experimental paradigms typically involve structured tasks or experiments designed to measure specific cognitive processes or abilities, which help researchers gain insights into the impact of high‐altitude conditions on cognitive function and better understand the cognitive changes or challenges that individuals may experience at higher altitudes. These tests were chosen with the intention of assessing various cognitive domains or areas of cognitive functioning. Among the cognitive functions, memory, attention, executive function, and motor skills and construction, these specific cognitive domains have received substantial attention and interest from numerous researchers. But cognitive abilities are interconnected and interdependent, and testing them requires considering the interplay of various cognitive functions. The interpretation of a single test can vary depending on researchers' theoretical preferences and the specific experimental context. Different researchers may interpret the same test as measuring different cognitive functions based on their theoretical perspectives or hypotheses. Additionally, the specific context in which the test is administered can influence how it is interpreted and the cognitive processes it is believed to assess. As a result of these factors, inconsistencies may arise in the findings of cognitive studies and contribute to the difficulties in selecting appropriate tests for assessing cognitive functions

Computerized cognitive assessment systems offer strict control over stimulus presentation and precise measurement of response time, which helps minimize operational and recording errors resulting from subjective factors of the examiner. As a result, these systems enhance the standardization of cognitive tests, ensuring more reliable and consistent results. These systems can be implemented using various digital interfaces such as computers, tablets, and handheld devices. This allows for efficient testing of a large number of individuals, providing benefits such as on‐demand assessment, accurate measurement of cognitive abilities, shorter testing duration, easy administration in different languages, automated data export, minimal requirement for professional involvement, automated result calculation, and generation of descriptive reports. However, the sentence acknowledges some limitations of computerized cognitive assessment systems. It mentions that these systems often require a relatively long testing duration compared to other assessment methods. Additionally, different platforms may require specific software and hardware environments, which can be a constraint. The use of specialized custom hardware may limit the applicability of these systems to certain scenarios. Furthermore, the dependence on commercial companies for most computerized cognitive assessment systems may restrict their usage to specific contexts, potentially limiting their accessibility or availability in certain settings. Although computerized cognitive assessment systems have some disadvantages, it provides a high level of accuracy and precision, making them suitable and effective for most cognitive screening efforts. In other words, using computer‐based assessments can be beneficial for evaluating cognitive functions because of their ability to generate reliable and precise measurements, which can aid in identifying cognitive impairments or changes more accurately than traditional paper‐and‐pencil assessments.

### 4.2 Suggestions for choosing an appropriate cognitive assessment method

Based on our review, the wide variation in factors such as exposure altitude, length of exposure, rate of ascent, and other experimental conditions at high altitudes makes uniform comparison across the numerous assessment methods difficult. For example, nine studies (Griva et al., 2017, Altbäcker et al., 2019; Asmaro et al., 2013; Chroboczek et al., 2021; De Aquino et al., 2012; Issa et al., 2016; Latshang et al., 2013; Lefferts et al., 2020; Ochi et al., 2018; Pun et al., 2018; Sharma et al., 2014) investigate the effect of altitude by Stroop Test, but results were highly varied or diverse, showing considerable differences depending on the specific test being used and the altitude levels being examined. Thus, determining the optimal assessment methods remains challenging. However, we can facilitate selection of appropriate assessment tools by considering the following key aspects.

#### 4.2.1 Altitude and time duration of exposure

The degree of cognitive impairments corresponds to the height attained during high‐altitude ascent. Researcher (Yan, 2014) has demonstrated that greater altitude is associated with more severe impairments in cognitive function. Some assessment methods may not be sensitive to relatively low altitudes. When choosing cognitive assessment tools, examining studies that employed comparable altitude conditions to one's own experiment may prove beneficial.

Another factor is time duration of exposure. The effects of high‐altitude environments on physical and mental states are dynamic, changing with both increased elevation and prolonged duration at altitude. When people enter this hypoxia environment, there are three stages of adaptation to high altitude: the acute period within the first 3 days of altitude exposure, the subacute period from 3 days to 1 month, and the chronic period beyond 1 month (Zubieta‐Calleja et al., 2011). Cognitive function parallels physiological adaptation to high altitude, with the greatest declines observed initially upon exposure. While extended time at altitude led to hypoxic acclimatization and some recovery of cognitive function, performance remained below that of control subjects at sea level (Zhu & Fan, 2017). Especially for exploring the long‐term effects of high‐altitude environments on cognitive function, in addition to using neuropsychological tests, electrophysiology test, and neuroimaging technique should be considered.

#### 4.2.2 Ascent mode

The method of ascending to altitude (such as climbing, trekking, or driving) may impact performance on neurocognitive tests administered at high elevations. Passive ascent by car or cable car typically involves faster ascent rates than active ascent by foot. As previously described, a more gradual rate of ascent allows for improved acclimatization to high‐altitude environments. As described above, good acclimatization can relatively alleviate the effects of high‐altitude environments on cognitive function, and be given to the rate at which altitude exposure occurs.

In fact, the rate of ascent is a factor that is related to the type of research, namely, laboratory research versus field research. In laboratory assessments, hypoxic conditions were often induced rapidly rather than following the more gradual pace of active ascent in field investigations.

#### 4.2.3 Practice effect

The factors discussed above are all influences of experimental conditions on the selection of assessment methods. In fact, not only experimental conditions, but also the practice effect of the assessment methods themselves, are factors that need to be considered when selecting appropriate assessment methods. In a test–retest design, the potential for measurement errors and practice effects to artificially inflate a patient's true score exists (Lemay et al., 2004). When making a selection for a test or assessment, it is advantageous to opt for a test that is not highly influenced by practice effects;

Another way to reduce this practice effect is through extensive practice. By using these extensive familiarization trials, researchers or practitioners attempted to prepare participants in a way that their performance would remain relatively stable and consistent during the actual assessments.

Alternatively, by using parallel versions or alternative forms of tests, individuals are less likely to experience performance improvements due to familiarity. These versions or forms are designed to be equivalent in terms of content and difficulty but are different from the original test, so participants cannot rely on previous exposure to the test to boost their scores. This approach aims to obtain more accurate and unbiased assessments of an individual's cognitive abilities.

## 5 LIMITATION AND CONCLUSION

During the course of conducting this review, several limitations were identified. One limitation was the lack of detailed reporting in many of the included studies. Specifically, many of these studies did not provide specific data such as performance scores from the cognitive tasks. Second, this is narrative review, which offers a broad perspective and interpretation of existing literature without following a strict methodology, while a systematic review employs a rigorous and transparent approach to identify, analyze, and synthesize evidence to answer a specific research question. Systematic reviews provide more robust and reliable conclusions compared to narrative reviews due to their systematic and objective methodology. Third, although our review covers studies included acute exposure, chronic exposure, laboratory study, field study, in order to understand the effect of pure high‐altitude environment on cognition, choosing the suitable assessment approach, preacclimation study will be missing. Last, while the nature of most cognitive domains is generally accepted, there are still noticeable discrepancies in the clinical and research literature. These inconsistencies primarily arise in broad domains that might encompass several component processes. Determining whether these processes should be classified under more general domains (e.g., executive functioning) or simpler domains (e.g., processing speed) is often unclear and subject to different interpretations. As a result, there is a lack of consensus on how to precisely categorize certain cognitive processes within specific domains. So when grouping the test procedures into different cognitive domains, the division, or categorization was done subjectively, without clear and objective criteria that can be consistently replicated by others. In other words, the categorization was based on subjective judgment rather than on standardized or universally agreed‐upon criteria. As a result, there may be some ambiguity or lack of consistency in how the tests were grouped into specific cognitive domains.

For the increase in exposure to high‐altitude hypoxic environments and the need for cognitive function evaluation, this review systematically organizes and summarizes the current cognitive function assessment methods in high‐altitude hypoxic environments, categorizing these methods roughly based on their different formats and the cognitive domains they evaluate. In the future, efforts are being made to develop a collectively recognized open‐source test system for high‐altitude cognitive assessment.

## AUTHOR CONTRIBUTIONS

**Haojie Fan**: Data curation; investigation; writing—review and editing. **Ying Meng**: Data curation; writing—review and editing; investigation. **Lingling Zhu**: Supervision; resources. **Ming Fan**: Writing—review and editing; resources. **Du‐Ming Wang**: Writing—review and editing; resources. **Yong‐Qi Zhao**: Writing—review and editing; funding acquisition; resources.

## FUNDING

The authors received no funding for this work.

## CONFLICT OF INTEREST STATEMENT

All authors agree on the final expression of the manuscript, and there are no conflicts of interest between them.

### PEER REVIEW

The peer review history for this article is available at https://publons.com/publon/10.1002/brb3.3418.

## Supporting information

Supplementary Information

Table S1. View of the studies examining the effect of altitude and hypoxia on cognition.

## Data Availability

All data used in this review were from PubMed, and are available from the corresponding author upon reasonable request.
