# Supplementary material for: A review of methods for assessment of cognitive function in high‐altitude hypoxic environments
Source: Brain Behav. 2024 Feb 26;14(2):e3418. doi: 10.1002/brb3.3418 (PMC10897364; doi:10.1002/brb3.3418)
Supplement: Supplementary file 1 — Supplementary Information [file BRB3-14-e3418-s001.docx]

Hypoxia exposure, acute or chronic, will cause varying degrees of damage to the cognitive function of the brain, and assessment and prevention of such cognitive impairment would facilitate detection of cognitive changes and impairment. So, this paper summarizes the findings of previous research, outlines the methods for cognitive function assessment, elaborates and systematic cognitive function assessment tools for high altitude hypoxia environments, and predicts the prospect of research and development.
