# Supplementary material for: A review of methods for assessment of cognitive function in high‐altitude hypoxic environments
Source: Brain Behav. 2024 Feb 26;14(2):e3418. doi: 10.1002/brb3.3418 (PMC10897364; doi:10.1002/brb3.3418)
Supplement: Supplementary file 2 — Table S1. View of the studies examining the effect of altitude and hypoxia on cognition. [file BRB3-14-e3418-s002.docx]

**Table S1. View of the Studies Examining the Effect of Altitude and Hypoxia on cognition.**

| **Reference (First authors)** | **Participants** | **Intervention** | **Control/Comparison** | **Cognitive tests** | **Impact of Altitude** |
| --- | --- | --- | --- | --- | --- |
| **Abraini (1998)** | 8 male climbers | 0m-8848 m,  hypobaric chamber, 31 days | baseline test at sea level control group at sea level | Pegboard‐Psychomotor Test  Number Ordination Rey’s Test  Visual Choice Reaction Time | Pegboard Psychomotor Test ↓ At 8,000 m (compared to control group), ↓At 8,848 m (compared to baseline) Number Ordination Rey’s Test  ↑Until 6,500 m (compared to baseline test) ↓ At 8,000 & 8,848 m (compared to control group)  ↔Visual Choice Reaction Time |
| **Altbäcker (2019)** | 12 climbers 10 male, 2 female | 5500m, normobaric (mixture gas) ,43 min | baseline test under normoxia | Modified Continuous Performance Task  Number‐Size Stroop Variant Task | ↔no significant change |

| **Reference (First authors)** | **Participants** | **Intervention** | **Control/Comparison** | **Cognitive tests** | **Impact of Altitude** |
| --- | --- | --- | --- | --- | --- |
| **Asmaro(2013)** | 35 volunteers 31 male, 4 female | hypobaric chamber, 5 min at 7620m, 30 min at 5334 m | baseline test conducted before entry to chamber | Digit Span Forward Task1 (DST‐F)  DS Backward Task1 (DST‐B)  Trailmaking A Task Trailmaking B Task  Stroop Task | Stroop Task ↓Accuracy ↓reaction times at 7,620 m DST‐F at 7,620 m & DST‐B at 5,334 & 7,620 m ↓ Correct responses  Trailmaking A and B Task  ↑completion time at 5334 and 7620m |
| **Bonnon (1999)** | 7 climbers 4 male, 3 female | 3500m-6200m,30 days, field study, sea level to 3,500 m (flight)  3500m to 6,200 m (climbing),  test at 3500 m and 5400m | baseline test under normoxia control group under normoxia | Cognitive Motor Task | ↔Cognitive Motor Task  compared to control group |

| **Reference (First authors)** | **Participants** | **Intervention** | **Control/Comparison** | **Cognitive tests** | **Impact of Altitude** |
| --- | --- | --- | --- | --- | --- |
| **Chen(2017)** | 49 college students 32 male 17 female | 3,658 m, 2 years , field study | baseline test at 446m | CNS Vital Signs: verbal memory test, visual memory test, simple reaction time test, go/no‐go test | ↓Accuracy in Memory Test ↑Responding Time in reaction time test ↑reaction time in go/no‐go test |
| **Chroboczek(2021)** | 15 physically active men | 3500m,normobaric(mask), 30min | baseline test under normoxia | Stroop Interference Test | ↑ reaction time and time delta of "naming" stage. |
| **Crow (1971)** | 86 medical students 60 male 26 female | 2,438 m and 3,656 m,30 min hypobaric chamber | baseline test at  under normoxia | Number recall task | ↔ Number recall task |
| **Davranche (2016)** | 11 male climbers | 4350m,4 days, field study cognitive assessment conducted on Day 1, Day 2, and Day 4 | baseline test  at sea level | Simon Task  Time Perception Task | Simon Task ↑Reaction time on Day 1 ↑ Errors on Days 1, 2, and 4 on congruent trials Time Perception Task ↓durations |

| **Reference (First authors)** | **Participants** | **Intervention** | **Control/Comparison** | **Cognitive tests** | **Impact of Altitude** |
| --- | --- | --- | --- | --- | --- |
| **De Aquino Lemos (2012)** | 10 male participants | 4,500 m,24 h normobaric chamber | Normobaric chamber at sea level | DST-F DST-B Sequences of Numbers and letters  Corsi Blocks Forward  and Backward  Random Number Generation  Stroop Color and Word  Test | ↓Digit Span  Test Forward  ↓Digit Span Test Backwards  ↓Sequence of Numbers  and Letters Test  ↓ Corsi Block Tests  ↓ Random Number  Generator Test  ↓ Stroop Color Test |
| **De Bels (2019)** | 17 male participants | 0-3842m,4 h with ascent and descent, hypobaric chamber, test at 3842m | baseline test  at sea level | Modified Math Processing Task , Perceptual Vigilance Task , Time Wall Estimation Task | ↔No change all three tests |
| **Dykiert (2010)** | 10 participants 6 male, 4 female | 1992-5565m,20 days, field study, 1,992 m to 3,271 m(bus) ,3271m-5565m(trekking), 21 assessments | baseline test  at 1992m | Choice reaction time | ↓reaction time above 4,000 m revealed in linear mixed modeling |

| **Reference (First authors)** | **Participants** | **Intervention** | **Control/Comparison** | **Cognitive tests** | **Impact of Altitude** |
| --- | --- | --- | --- | --- | --- |
| **Falla (2019)** | 36 participants 18 male, 18 female | 1258m-3269m,3 days field study 1258-2178m(climbing) 2178-3269m(driving) test at 3269m | baseline test at 1258m above sea level | Digit Symbol Substitution Test (DSST)  Psychomotor Vigilance Test(PVT) | DSST ↓correct trials(day2)  PVT ↓reaction time BART ↑mean pumps |
| **Frost (2021)** | 15 participants 10 male, 5 female | 3 days, field study 340 m to 1216 m in 4 h 1,216 m to3,800 m in 2 h 3 tests at 3800m in 3 days | baseline test at 340m above sea level | PVT  BART  DSST Line Orientation Task N‐Back Task  Visual Object Learning Task  Abstract Matching Task Motor Praxis Task | PVT  ↑reaction times(day3) BART  Faster reaction times (day2 and day3)  ↔Number of pumps  ↔DSST, Line Orientation Task, N‐Back Task, Abstract Matching, Motor Praxis Task |
| **Gibbons(2020)** | 10 male participants | 16 ± 4 days,field study, from sea level to 4330m in 6 h(car) | baseline test at 344m above sea level | Pro‐point and Anti‐Point Tasks | ↔no change |

| **Reference (First authors)** | **Participants** | **Intervention** | **Control/Comparison** | **Cognitive tests** | **Impact of Altitude** |
| --- | --- | --- | --- | --- | --- |
| **Griva (2017)** | 198 participants 60% male,40% female | 3500m-5300m,11 days, field study(climbing), test at 3500m and 5300m | baseline test at 75 m above sea level control group at/or near sea level | Trail making A Task, Trail making B Task,  Controlled Oral Word Association Test,  Letter Cancellation Test, Stroop Test,  Grooved Pegboard,  Rey Auditory Verbal Learning Test,  Symbol Digit Modalities Test,  Block Design Test | ↓decline in all tasks |
| **Harris(2009)** | 26 volunteers 15 male, 11 female | 410 m up to 5,400m ,18 days, field study(climbing), test at 5400m | baseline test at sea level | DST‐F DST‐B DSST Trail‐making Test B  Reyʹs Auditory‐Verbal Learning Test Controlled Oral Word Association Test | ↓Reaction time on CogState monitoring task ↓ Correct responses on DST‐F ↑ Correct responses on DSST |

| **Reference (First authors)** | **Participants** | **Intervention** | **Control/Comparison** | **Cognitive tests** | **Impact of Altitude** |
| --- | --- | --- | --- | --- | --- |
|  |  |  |  | CogState:  Simple Reaction Time  Choice Reaction Time,  Monitoring Task Reaction Time,  Monitoring Task Accuracy,  Working Memory Task Reaction Time,  Working Memory Task Accuracy, Learning Task Accuracy | ↓ Time to completion on trial making task B  ↔ other tasks |
| **Issa (2016)** | 8 volunteers | 1400-5000m,23 days, field study, trekking, test at 5000m | Cognitive assessment conducted at Sea level | Rapid Cognitive Assessment Tool  Stroop Color‐Word test Trail Making test A and B | ↔No significant changes |
| **Karinen (2017)** | 9 male climbers | 1400m-8848m,80 days, field study(climbing), assessment conducted at 5,300 m on Days 16, 34, 43, and 62 | Cognitive assessment conducted at 1,400 m | Colorado Perceptual Speed Test Number Comparison Test | ↑ Errors on perceptual speed test ↔Number Comparison Test |

| **Reference (First authors)** | **Participants** | **Intervention** | **Control/Comparison** | **Cognitive tests** | **Impact of Altitude** |
| --- | --- | --- | --- | --- | --- |
| **Kramer(1993)** | 40 volunteers 36 male, 4 female 20 climbers, 20 controls | 18 to 26 days, field study, from 2,195 m up to 6194 m(Expedition) | control group test at sea level | Automated Performance Test System (APTS) battery: Pattern Comparison Task  Code Substitution Task  Choice Reaction Time Task  Memory Search Task Finger‐Tapping Task Category Search Task | ↓ Slow performance with higher reaction and response times compared to CG |
| **Latshang (2013)** | 51 male volunteers | 4 consecutive days at 1,630 m and 2,590 m, 2 days each, field study, cable car, test at 1630m and 2590m | baseline test at 490m  above sea level | Psychomotor Vigilance Test, Divided Attention Steering Simulator, Number Back Task,  Trail Making Test A | ↔no significant changes |
| **Lefferts (2019)** | 18 volunteers  10 male 8 female | 1400 m-5160 m,11 days, field study(climbing), | Cognitive assessment  conducted at 116m above sea level | Flanker Task  N‐Back Number Task | Flanker Task  ↓Faster RT at 4,240 m  N‐Back Number Task  ↓accuracy at 5,160 m |

| **Reference (First authors)** | **Participants** | **Intervention** | **Control/Comparison** | **Cognitive tests** | **Impact of Altitude** |
| --- | --- | --- | --- | --- | --- |
| **Lefferts(2020)** | 8 volunteers 4 male 4 female | 1400m to 4240m, 7 days, field study(trekking) test at 3440m and 4240m | Cognitive assessment conducted at 1400m | Stroop Task | ↑Accuracy at 4,240 m  ↓reaction times at 3,440 m and 4,240 m |
| **Limmer(2018)** | 25 volunteers 19 male, 6 female control group: 21 volunteers,11 male,10 female | 3500m and 5800m, each altitude 21min, normobaric chamber | Cognitive assessment conducted under normoxia | Frankfurt Attention Inventory‐2  Performance Value, Continuity Value and Quality Value | ↓Performance Value, Continuity Value and Quality Value at 5,800 m compared to CG |
| **Loprinzi (2019)** | 21 volunteers 10 male ,11 female | 4000m,30min,normobaric(mixture gas) | control group test under normoxia | Memory Interference Task | ↑Retroactive interference for immediate and delayed  ↔Proactive interference |
| **Ma(2019)** | 19 college students 10 male,9 female control group:40 college students, 19 male,21 female | 3650m, 3 years,field study | control group test at sea level | n-back tasks (verbal and spatial) | ↓ Response accuracy in both the verbal and spatial 2-back tasks. |

| **Reference (First authors)** | **Participants** | **Intervention** | **Control/Comparison** | **Cognitive tests** | **Impact of Altitude** |
| --- | --- | --- | --- | --- | --- |
| **Ma(2016)** | 17 male college students | 5000m,less than 15 minutes, normobaric(mixture gas) | baseline test at sea level | mental rotation task | ↔response time  ↔error rate. |
| **Malle(2016)** | 4 male climbers | 1400m-8848m,6 weeks,1,00 m to 5,500 m(13 days,trek) 5500m to 8043 m (4 weeks,trek),  14 assessments of PASAT between 1,400 m and 7,200 m  6 assessments of DST  subtests between 1,400 m and 5,600 m | baseline test at sea level | Paced auditory serial addition test (PASAT) DST‐F  DST‐B | ↔No significant change in any of the three cognitive tasks |
| **Merz (2013)** | 32 mountaineers 25 male,7 female | 4,497 m, 5,533m, and 6,265 m, 21 days, field study trekking, | Cognitive assessment conducted prior to expedition at 440m | Line Bisection Test  Ruff 2/7 Cancellation Test  Ruff Figural Fluency Test (RFFT)  Modified Pegboard, Chess test | Ruff 2/7 Cancellation Test ↓ Time to completion from 440 m to 6,265 m |

| **Reference (First authors)** | **Participants** | **Intervention** | **Control/Comparison** | **Cognitive tests** | **Impact of Altitude** |
| --- | --- | --- | --- | --- | --- |
| **Ochi(2018)** | 21 volunteers 14 male ,7 female | 16.5 min,2,000/3,500/5,000 m, normobaric(mixture gas) | baseline test at sea level | Color–Word Stroop Task | ↓ reaction times at 5,000 m  ↔Error rate Significant main effect of condition for reaction time in Stroop interference |
| **Parker (2017)** | 10 orthopedic surgeons 7 male, 3 female | 3,000 m,45 min  normobaric chamber | Cognitive assessment conducted in a normobaric chamber at 113 m | Verbal working memory task | ↔ Verbal working memory |
| **Pavlicek (2005)** | 21 male volunteers | group 1:450m,1500m,4500m,2 h, hypobaric chamber  group2:450,1500m,3000m,2 h, hypobaric chamber | 450-650m hypobaric chamber | Verbal Letter Fluency Task and three Category Fluency Task | ↔No significant changes |
| **Pelamatti (2003)** | 15 mountaineers | 4500 m -5050 m during different expeditions,3-5 days,field study(trekking), test at 4500m and 5050m | test at sea level | Verbal Free Recall | ↓Correct responses at altitude |

| **Reference (First authors)** | **Participants** | **Intervention** | **Control/Comparison** | **Cognitive tests** | **Impact of Altitude** |
| --- | --- | --- | --- | --- | --- |
| **Petiet (1988)** | 8 female climbers | 5273m-6248m, 40 days, field study(climbing), test at 3658m,4511m,5273-6348m | Cognitive assessment conducted prior to expedition at Sea level | Gorhamʹs Proverbs Paced Auditory Serial Addition Test  Digit Span Test  Finger Tapping  Selective Auditory Attention Task  Selective Reminding Test  Benton Line Orientation Task  Boston Naming Test | ↑Paced Auditory Serial Addition Test  Boston Naming Test ↓ Expressive language ability |
| **Phillips (1966)** | 18 male volunteers | 3800m, 2 days , field study | Cognitive assessment conducted at sea level | DST-F Word Span Forward Robinsonʹs Rhymes and Numbers tests | ↔No significant changes |
| **Pighin (2012)** | 30 students 14 male,16 female | 3000m, normoxic chamber, 70min | baseline test under  normoxia | Psychomotor Speed Task The financial  decision-making task | Psychomotor Speed Task ↑response times The financial  decision-making task |

| **Reference (First authors)** | **Participants** | **Intervention** | **Control/Comparison** | **Cognitive tests** | **Impact of Altitude** |
| --- | --- | --- | --- | --- | --- |
|  |  |  |  |  | ↑more risk seeking in the hypoxia for choices involving losses |
| **Pighin (2014)** | 26 university students 12 male, 14 female | 3,000 m,Normobaric chamber | Cognitive assessment conducted at sea level | Decision-making task | ↓loss aversion on decision-making task |
| **Pighin (2020)** | 26 volunteers 12 male,14 female | 3000m, normobaric chamber | baseline test under  normoxia | BART task | ↑ Adverse risk‐taking behaviour, higher num‐ ber of pumps |
| **Pramsohler (2017)** | 11 university students 6 male, 5 female | 3,500,5,500 m,2 nights  normobaric chamber | test conducted after 1 night at 450 m | Choice reaction test | ↑ Reaction time follow by increasing altitude |
| **Pun,Guadagni(2018)** | 21 volunteers 8 male,13 female | 520m-5,050 m, sleeping height 2,900 m and 6 days at 5050m, field study(motor vehicle) test at day1 and day6 at 5050m | baseline test at 502m | CANTAB: Reaction Time Task Attention Switching Task Rapid Visual Processing One Touch Stockings of Cambridge Task | ↑Selective and sustained attention improves with acclimatization |

| **Reference (First authors)** | **Participants** | **Intervention** | **Control/Comparison** | **Cognitive tests** | **Impact of Altitude** |
| --- | --- | --- | --- | --- | --- |
| **Pun, Hartmann(2018)** | 21 volunteers 8 male,13 female | 520m-5,050 m, sleeping height 2,900 m and 6 days at 5050m,  field study(motor vehicle)  test at day1 and day6 at 5050m | baseline test at 502m | PVT  Trail Making Tests A &B | PVT  ↑reaction times at acute exposure to 5,050 m |
| **Roach(2014)** | 21 participants  12 male,9 female | 1525m-5260m,16days,field study(driving) conducted after 1 and 16 days at 5,260 m | baseline test at sea level ,1 month before traveling | Simple Reaction Time | ↓ decrease in throughput dSRT with acute altitude exposure |
| **Schlaepfer(1992)** | 10 participants  6 male,4 female | 1. 3450m,25min, field study, trekking 2. 3450m, 25min, normobaric mask | Cognitive assessment conducted at 540m | Time Needed Reading Briefly Displayed Letters | ↓ Time to completion in both conditions |
| **Seo (2015)** | 16 male participants | 2 hours, normobaric chamber,4300m | baseline test under  normoxia | ANAM subtests: Go/No‐Go Test Running Memory Continuous Performance test | Memory Continuous Performance test ↓ less correct response and throughput score |

| **Reference (First authors)** | **Participants** | **Intervention** | **Control/Comparison** | **Cognitive tests** | **Impact of Altitude** |
| --- | --- | --- | --- | --- | --- |
| **Seo (2017)** | 16 female participants | 2 hours, normobaric chamber,4300m | baseline test under  normoxia | ANAM subtests:  Memory Continuous Performance test | Total Mood Disturbance ↓ worse score |
| **Sharma(2014)** | 850 male volunteers | Assessment conducted after 3, 12, and 18 months of living at 4,200–4,600 m | Baseline test at 240m | MCI Screening Tests  Raven’s progressive matrices  Clock Drawing Test  Bender Visual Motor Gestalt Test  Serial digit learning test  Stroop task  Trail making task  Verbal Fluency Test | ↔no significant change |
| **Shi (2016)** | 30 male volunteers | 4280m,3h, field study sport utility vehicle | baseline test at 490m | Visual digit-span test  Auditory digit-span test  Paced Visual Serial Addition Test  Paced Auditory Serial Addition Test  Picture Recall Test Picture Recognition Test | ↓Auditory digit-span test ,  ↓Paced Visual Serial Addition Test ,  ↓Paced Auditory Serial Addition Test ↓ Picture Recognition Test |

| **Reference (First authors)** | **Participants** | **Intervention** | **Control/Comparison** | **Cognitive tests** | **Impact of Altitude** |
| --- | --- | --- | --- | --- | --- |
| **Stepanek (2013)** | 25 participants  14 male,11 female | 5min,7,101m,normobaric(mixture gas) | baseline test under  normoxia | King‐Devick Test | ↓slower completion time |
| **Subudhi (2014)** | 21 participants  12 male,9 female | 1525m-5260m,16days,field study(driving) ognitive assessment conducted after 1 and 16 days at 5,260 m | baseline test at sea level ,1 month before traveling | DANA test  SRT1 & SRT2  Procedural Reaction  Time  Go/No‐Go  Code Substitution simultaneous  Code Substitution ‐delayed recall  Spatial Discrimination  Match to Sample  Sternberg’s Memory  Search | ↑ Simple reaction time Day 1, improved by Day 16 ↑ Choice reaction time Day 1, improved by Day 16  ↓ Code substitution Day 1, improved by  Day 16  ↓ Match to sample Day 1, improved by Day 16 ↔ Code substitution  delayed, go/no-go or memory search |
| **Thomas(2007）** | 11 volunteers 7 male, 4 female | 9 nights，2020m, normobaric tent, Cognitive assessment conducted on Days 4, 8, 9, and 15 | Cognitive assessment conducted at sea level | Psychomotor vigilance task 2-back task | ↔No significant changes |

| **Reference (First authors)** | **Participants** | **Intervention** | **Control/Comparison** | **Cognitive tests** | **Impact of Altitude** |
| --- | --- | --- | --- | --- | --- |
| **Turner(2015)** | 22 volunteers 10 male, 12 female | 90min,5500m, normobaric(mixture gas) | baseline test under  normoxia | CNS Vital Signs: Verbal and Visual  symbol digit substitution,  Memory Finger Tapping  Digit Symbol Substitution Test  Stroop Test  Shifting Attention  Continuous Performance Test | ↓ Scores on verbal memory, visual memory  Stroop test, shifting attention  and continuous performance test |
| **Weigle(2007)** | 19 volunteers 10 male, 9 female | 1,200 – 4,300 m,12 days, field study, Cognitive assessment conducted on Days3 and Day6 at 3810m | baseline test at sea level | Visual Motor Reaction Time Stroop Color‐Word Test Verbal Reasoning Test: Sentence Repetition | Visual Motor Reaction Time ↓ slower at 3,810 m Stroop Test ↑longer completion time |
| **Williams(2019)** | 11 male volunteers | 600m,3000m,4500m,1h each altitude,  normobaric chamber | Normobaric chamber at sea level | Eriksen flanker N‐Back Number Task Deary–Liewald Reaction Time Task | N‐Back Task  ↓accuracy at 3000m(compare sea level,1600m) |

| **Reference (First authors)** | **Participants** | **Intervention** | **Control/Comparison** | **Cognitive tests** | **Impact of Altitude** |
| --- | --- | --- | --- | --- | --- |
|  |  |  |  |  | ↓accuracy at 4500m(sea level) |
| **Xin(2020)** | 49 college students 32 male 17 female | 3650m, 2 year, field study | baseline test at 400m above sea level | CNS Vital Signs: verbal memory test, visual memory test, simple reaction time test, go/no‐go test | ↓Accuracy in Memory Test ↑Responding Time in reaction time test  ↑reaction time in go/no‐go test |
| **Zhang (2018)** | 20 volunteers 10 male ,10 female | 3680m,3 years, field study | control group test at  46m above sea level | visual search task | ↑reaction times |
| **Zhang(2013)** | 47 male volunteers | 3,700m, 5 days, field study, | baseline test at 300 m | Neurobehavioral core test battery: Simple Reaction Time Test  DST‐F and DST‐B Santa Ana Manual Dexterity Test  DSST  Benton Visual Retention Test  Pursuit Aiming Test | ↓Santa Ana Manual Dexterity Test ↓DSST, ↓Pursuit Aiming Test |

| **Reference (First authors)** | **Participants** | **Intervention** | **Control/Comparison** | **Cognitive tests** | **Impact of Altitude** |
| --- | --- | --- | --- | --- | --- |
| **Zhang(2011)** | 52 students 24 male,28 female | 2260m,7-month, field study | control group test at sea level | Wechsler Memory Scale tests, verbal and spatial 2-back working memory tests,  long-term explicit memory tests, Rey Auditory Verbal Learning test,  Rey–Osterrieth Complex Figure test, degraded picture naming test,  Serial Reaction Time Test | ↓short-term visual construction ↓Rey–Osterrieth Complex Figure test |
| **Wang(2021)** | 71 students  33male,38 female | 3660m,3years,field study | control group test at sea level | two-choice oddball task | HA group yielding lower accuracy |
| **Ma(2019)** | 52 Tibetan students | 2,700 m, 3,700 m, and 4,500 m, field study | 2700m | flanker task | not significant at a behavioral level |
